# Optimal Coordination Strategy for International Production Planning and Pollution Abating under Cap-and-Trade Regulations

**DOI:** 10.3390/ijerph16183490

**Published:** 2019-09-19

**Authors:** Baogui Xin, Wei Peng, Minghe Sun

**Affiliations:** 1 Nonlinear Science Center, College of Economics and Management, Shandong University of Science and Technology, Qingdao 266590, China; 2 Department of Management Science and Statistics, College of Business, The University of Texas at San Antonio, San Antonio, TX 78249-0634, USA

**Keywords:** optimal coordination strategy, carbon emission, production plan, pollution abatement, differential game

## Abstract

Because both pollution emissions and production policies often are international in scope, it is necessary to find optimal coordination strategies for international production planning and pollution abating. Differential game models are developed for multiple neighboring countries to reach optimal decisions on their production planning and pollution abating under cap-and-trade regulations. Non-cooperative and cooperative differential games are presented to depict the optimal tradeoffs between production planning and pollution abating. Hamilton-Jacobi-Bellman (HJB) equations are then employed to analyze the asymmetric and symmetric feedback solutions. Numerical simulations are used to illustrate the results. Five different dividends are also discussed. With the proposed strategies, more improvement will be directed toward production supplies and environmental issues than ever before.

## 1. Introduction

Since the middle of the 20th century, the rapid population growth and economic development have led to serious environmental pollution that has critical impacts on crop yields, climate change, biodiversity, and ecosystems, amongst others. As a result, these impacts have significant social and economic consequences affecting values, customs, human health, economic development as well as social welfare [1]. In recent years, the growing concerns about industrial pollution, the strict regulations of international conventions on sustainability and environmental protection, and an increase in the number of pro-environmental consumers have led industries to dedicate significant efforts to developing green practices [2,3,4,5]. However, as of 2017, almost all advanced industrialized countries have had falling their emission rates, but still have not met their pledged emission reduction targets that they made in Paris [6]. Therefore, it is entirely necessary to conduct studies in pollution abatement.

The Paris climate agreement, a new global agreement to combat climate change within the United Nations Framework Convention on Climate Change (UNFCCC), was signed in 2016. The agreement aims at holding global warming to well below 2 °C and to “pursue efforts” to limit it to 1.5 °C [7]. As of March 2019, 195 countries have signed the agreement, and 186 of which have submitted emission abatement pledges as nationally determined contributions. To meet the pledges, some instruments are needed to regulate and ultimately reduce the amount of pollution emitted into the atmosphere. One such instrument is the cap-and-trade mechanism. In recent years, several countries, including China, have implemented cap-and-trade programs to set a national limit on carbon emissions by the heavy-polluting industries such as power generation, iron and steel, and chemicals, amongst others [8]. Firms must have enough permits to cover their emissions if they exceed the cap. Generally, firms can invest in clean technologies to become more efficient, switch to lower carbon fuels or purchase additional permits. Indeed, they may sell some permits if they do not use up the cap. By comparison with pollution tax, the cap-and-trade mechanism creates a commodity out of the right to emit pollutants and allows the commodity to be traded on the free market. However, there exist several dilemmas, such as a firm’s higher income depends on more output which may lead to more pollution emissions, and fewer permit purchases or more permit sales depend on greater abatement efforts which may lead to profit losses [9]. Thus this study deals with real demand for production planning and pollution abating under cap-and-trade regulations.

Pollution emissions often show the feature of being international, that is, there exists transboundary pollution which is a typical example of a negative externality and is also an essential instance of global environmental agreement failure [10]. When a negative externality occurs in neighboring countries, a country does not take responsibility for external costs but passes them on to others. Thus the producing country has lower marginal costs than it would otherwise have and may choose to produce more of the product than it would when it was required to pay all associated pollution costs. Hence, some governance mechanisms could be designed to “internalize” an externality. One such mechanism is to employ a differential game to optimize their production planning and pollution abating under cap-and-trade regulations.

Given the concerns mentioned above, the key contribution of this study is on developing differential game models for multiple neighboring countries to reach optimal decisions by optimizing their production planning and pollution abating under cap-and-trade regulations. By using Hamilton-Jacobi-Bellman (HJB) equations, the impacts of the production efficiency and the initial allocations of emission permits on the optimal production plans and the pollution abating decisions can be determined by analyzing the feedback equilibria of the proposed models.

The remainder of this paper is organized as follows. The relevant literature is reviewed in Section 2. In Section 3, differential game models are proposed for multiple neighboring countries. Utilizing the HJB equations, the non-cooperative differential games are studied in Section 4, and the cooperative differential games are discussed in Section 5. In Section 6, numerical simulations are performed to verify the results. In Section 7, the cooperative and symmetric dividends are defined, and the evolutions are analyzed by using numerical methods. In Section 8, the potential policies and managerial implications are discussed. Finally, a summary and future research directions are presented in Section 9.

## 2. Literature Review

As a powerful analytical tool, the differential game has been widely used in many scientific fields. You, Jiang and Li [11] employed the differential game to design optimal coordination strategies of regional emission abatement collaboration. Feng and Liu [12] used the differential game to consider a single supply chain structure concerning the online word-of-mouth effect. Schüller, Staňková and Thuijsman [13] put forward a differential game of pollution control while there are transboundary environmental effects of national policies. Di Liddo [14] set up a differential game to coordinate a pharmaceutical company controlling the drug price and a social planner determining the number of people to treat.

Many researchers have made significant efforts in searching for optimal strategies of pollution control, as shown in Table 1. Ploeg and Zeeuw [15] proposed a model of international pollution control. Yeung [16] employed a cooperative differential game to set up a model of transboundary industrial pollution control. He and Hua [17] studied transboundary pollution control strategies about production quantity, pollution taxes and abatement investment. Bertinelli, Camacho and Zou [18] proposed strategies to motivate business efforts to reduce CO_2_ emissions through a capture and storage mechanism. This study employs differential games to balance optimally business production planning and pollution abating to optimize income.

Transboundary/international pollution emission and abatement are now becoming one of the hottest topics in scientific research. A differential game is one of the powerful tools for studying transboundary pollution emission and abatement. Long [19] employed a differential game to depict the transnational pollution between two sovereigns, and analyzed its symmetric open-loop Nash equilibrium and Stackelberg leadership. Breton, Zaccour and Zaha [20] proposed a finite-horizon differential game model to analyze the joint implementation of environmental projects. List and Mason [21] developed an asymmetric differential game and determined the cooperative and non-cooperative equilibria. Yu and Xin [22] proposed a stochastic differential game to describe greenhouse gas (GHG) emission decision making of developed and developing countries. Yeung [16] presented a cooperative differential game of transboundary industrial pollution with industries and governments being separate entities and designed a payment distribution mechanism. Jørgensen [23] studied a differential game of waste management (disposal) between neighboring regions with strategic and stock externalities. Masoudi and Zaccour [24] considered a two-player differential game of international emissions to represent the interactions between developed and developing countries, and showed their cooperative and non-cooperative solutions. Wrzaczek, Shevkoplyas and Kostyunin [25] applied a differential game to formulate an overlapping generation model on optimal emissions with a continuous age structure. Bertinelli, Camacho and Zou [18] proposed a finite-horizon differential game to depict CO_2_ capture and storage and transboundary pollution and provided its explicit short-run dynamics with symmetric open-loop and a specific Markovian Nash strategy. Li [26] extended the model of Yeung [16] by taking into account emission permits trading, and found the optimal emission paths for the two regions. Gromova and Plekhanova [27] considered a differential game of transboundary pollution with developed and developing countries with a duration that is assumed to be exponentially distributed. Benchekroun and Martín-Herrán [28] studied a differential game of transboundary pollution between countries with myopic or non-myopic foresight. Huang, He and Hua [17] studied a cooperative differential game of transboundary industrial pollution between two asymmetric regions. Moreaux and Withagen [29] studied a differential game of carbon capture and storage from point sources with CO_2_ pollution and fossil fuel exhaustibility. Li [30] studied the behavior of the government about how to invest in emission abatement, how to regulate emission caps, and how to sell emission permits in a region divided into two economic sectors. Chang, Qin and Wang [31] involve the learning by doing in abatement in a transboundary pollution game. From a mathematical point of view, a transboundary pollution emission model can be used to approximately describe the transboundary GHG and other kinds of transboundary pollution emissions. 

Although at first glance there are many similarities in the literature mentioned above, there are always some subtle differences from an economic point of view, mainly reflected in their objective functions and stock dynamics equations of carbon or pollution. In this study, to depict the complex scenarios of coordination strategies for international production planning and pollution abating under cap-and-trade regulations, we try to integrate the pollutant stock level, the abatement effort, the production quantity, the initial allocation of pollution emission permit and the emission permit trading price into the objective function and stock dynamics equation. 

## 3. Modelling

All notations used through the paper are summarized in Table 2.

Although usually only two countries are involved in transboundary pollution emissions, a more general case is considered in this study. Assume n countries, labelled by i=1,2,⋯,n, compete as pollution emission oligopolies with production activities creating pollutant as an undesirable by-product, and share the same pollution state. They also optimize tradeoffs between production planning and pollution abating under cap-and-trade regulations. The pollutant emitted by the n countries is assumed to be identical. Furthermore, $e_{i}(t)$ is assumed to be proportional to $q_{i}(t)$, i.e.,

(1) $$e_{i}\left(q_{i}(t)\right)=\mu q_{i}(t).$$

The production quantity $q_{i}(t)$ can bring country i a certain level of net revenue, so that $R_{i}^{\prime }(t)$ can be written as

(2) $$R_{i}^{\prime }\left(q_{i}(t)\right)=\alpha_{i}e_{i}(t)-\frac{1}{2}e_{i}{\left(t\right)}^{2}=\alpha_{i}\mu q_{i}(t)-\frac{1}{2}\mu^{2}q_{i}{\left(t\right)}^{2}.$$

The production activity of each country generates not only net revenue but also a level of pollution with negative effects on its welfare. The environmental damage cost $D_{i}(s(t))$ is supposed to satisfy

(3) $$D_{i}(s(t))=\frac{1}{2}\beta_{i}s^{2}(t).$$

As we know, the higher the pollutant concentration is, the more costly the damage is to the country. The welfare of one country depends not only on its own policies but also on those of other countries [32]. However their excessive production activities can cause increasing environmental pollution, and even have negative impacts on their welfare levels. While one country bears the pollution abatement costs, all countries can share the benefits from any reduction in emissions. It is reasonable for each country to do its best to opt for a free ride. Hence, one of the critical things for country i is to decide how much effort it should make on pollution abatement. Suppose the pollution abatement cost function $G_{i}(u_{i}(t))$ of country i is quadratic and depends on the effort on pollution abatement of the country

(4) $$G_{i}(u_{i}(t))=\frac{1}{2}\sigma u_{i}{}^{2}(t),$$

which means that the more pollution abatement effort the country makes, the more costly the pollution abatement is to the country.

Analogous to human beings, a country’s abatement action also has egoistic and altruistic sides, i.e., the former acts for the country’s self-interest and directly benefits the country’s revenue, and the latter is helpful for the others. The total pollution abatement amount of country i, also divided into egoistic and altruistic parts, is supposed to be proportional to its abatement effort, denoted by $\delta u_{i}(t)$. The egoistic part of pollution abatement amount $B_{i}\left(u_{i}(t),s(t)\right)$ of country i is simultaneously determined by its abatement effort and the pollutant stock level, as shown in (5)

(5) $$B_{i}\left(u_{i}(t),s(t)\right)=\gamma_{i}u_{i}(t)s(t).$$

The trading revenue $R_{i}^{\prime \prime }\left(\cdot \right)$ of country i is written as

(6) $$R_{i}^{\prime \prime }\left(q_{i}(t),s(t),u_{i}(t)\right)=k\left(F_{i}+B_{i}\left(u_{i}(t),s(t)\right)-e_{i}\left(q_{i}(t)\right)\right)=k\left(F_{i}+\gamma u_{i}(t)s(t)-\mu q_{i}(t)\right).$$

Apparently, the following three cases holds: $R_{i}^{\prime \prime }\left(\cdot \right)>0$ if country i has permit surplus, and $R_{i}^{\prime \prime }\left(\cdot \right)=0$ if the permit of country i is just the right amount, and $R_{i}^{\prime \prime }\left(\cdot \right)<0$ if country i has permit shortage.

From (2)–(6), the instantaneous revenue of country i is given by (7) in the following

(7) $$\begin{array}{ll} \pi_{i}(t) & =\;R_{i}^{\prime }(q_{i}(t))+R_{i}^{\prime \prime }\left(q_{i}(t),s(t),u_{i}(t)\right)-G_{i}\left(u_{i}(t)\right)-D_{i}(s(t))\\ & =\mu \alpha_{i}q_{i}(t)+\;k\left(F_{i}+\gamma u_{i}(t)s(t)-\mu q_{i}(t)\right)-\frac{1}{2}\left(\mu^{2}q_{i}{\left(t\right)}^{2}+\;\beta_{i}s^{2}(t)+\;\sigma u_{i}{}^{2}(t)\right) \end{array}$$

The more goods the n countries produce, the more pollutants are emitted and, eventually, the higher the pollutant stock level is. However, each country can make its effort to decrease the pollutant stock level, and natural absorption or decomposition also reduces some pollutants. Suppose the decay function of pollutant stock is linear given by εs(t). Then the evolvement dynamics of the pollutant stock level is given by (8) in the following

(8) $$\begin{array}{ccc} \dot{s}(t)=\mu \sum_{i=1}^{n}q_{i}(t)-\delta \sum_{i=1}^{n}u_{i}(t)-\varepsilon s(t), & s\left(0\right)=s_{0}, & s\left(t\right)\geq 0 \end{array},$$

In a non-cooperative differential game, each country independently chooses its production plan and pollution abatement to maximize its own discounted infinite-horizon utility as stated in (9) in the following

(9) $$\begin{array}{cc} \underset{q_{i}\left(t\right),u_{i}\left(t\right)}{\max}J_{i}=\int_{0}^{\infty }e^{-rt}\pi_{i}(t)dt, & i=1,2,\cdots ,n \end{array},$$

subject to (8).

In a cooperative differential game, n countries make joint decisions of the production plan and pollution abatement controlled by a central authority, such as the European Union, to maximize the sum of the discounted infinite-horizon utilities of all countries as stated in (10) in the following

(10) $$\underset{q_{i}\left(t\right),u_{i}\left(t\right)}{\max}J=\int_{0}^{\infty }e^{-rt}\sum_{i=1}^{n}\pi_{i}(t)dt,$$

subject to (8).

In the following, NCA, NCS, CA and CS in the superscripts indicate the asymmetric non-cooperative, symmetric non-cooperative, asymmetric cooperative and symmetric cooperative differential games, respectively. For expositional convenience, time-dependence and superscripts will be omitted from the notations if no confusion is caused.

## 4. Non-Cooperative Differential Games

### 4.1. Asymmetric Case (NCA)

#### 4.1.1. Solutions of the Model

From (8) and (9), the HJB equation for country i is given in (11) in the following

(11) $$rV_{i}^{NCA}=\underset{q_{i},u_{i}}{\max}\left\{\pi_{i}+\frac{\partial V_{i}^{NCA}}{\partial s}\frac{ds(t)}{dt}\right\},$$

where $V_{i}^{NCA}=V_{i}^{NCA}\left(s\right)$ is the value function of country i.

The following optimal feedback production and abatement strategies of country i are obtained by taking first-order conditions of the HJB equation in (11) w.r.t. $q_{i}$ and $u_{i}$, respectively

(12) $$q_{i}^{NCA}\;=\;\frac{1}{\mu }\left(\alpha_{i}-k+\frac{\partial V_{i}^{NCA}}{\partial s}\right),$$

(13) $$u_{i}^{NCA}\;=\;\frac{1}{\sigma }\left(\gamma_{i}ks-\delta \frac{\partial V_{i}^{NCA}}{\partial s}\right).$$

Although the coefficients of the value functions in the following propositions can be explicitly characterized, for simplicity and regularity, they are characterized implicitly for all, including the symmetric, asymmetric, cooperative and non-cooperative, scenarios.

**Proposition** **1.** In the asymmetric non-cooperative differential game, the Nash equilibrium solutions of the value functions and instantaneous levels of production and abatement, represented by $V_{i}^{NCA}(s)$, $q_{i}^{NCA}(t)$ and $u_{i}^{NCA}(t)$, respectively, are

(14) $$V_{i}^{NCA}\left(s\right)=\eta_{i0}+\eta_{i1}s+\eta_{i2}s^{2},$$

(15) $$q_{i}^{NCA}\;=\;\frac{1}{\mu }\left(\alpha_{i}-k+\eta_{i1}+2\eta_{i2}s\right),$$

(16) $$u_{i}^{NCA}\;=\;\frac{1}{\sigma }\left(ks\gamma_{i}-\delta \left(\eta_{i1}+2\eta_{i2}s\right)\right),$$

where

(17) $$\eta_{i0}=\frac{1}{r}\left(\eta_{i1}\left(\sum_{j=1}^{n}\alpha_{j}-kn+\frac{\sigma +\delta^{2}}{\sigma }\left(\sum_{j=1}^{n}\eta_{j1}-\frac{\eta_{i1}}{2}\right)\right)+kF_{i}+\frac{1}{2}{\left(k-\alpha_{i}\right)}^{2}\right),$$

(18) $$\eta_{i1}=\frac{1}{r\sigma }\left(\begin{array}{l} 2\eta_{i2}\left(\left(\sigma +\delta^{2}\right)\left(\sum_{j=1}^{n}\eta_{j1}-\eta_{i1}\right)+\sigma \left(\sum_{j=1}^{n}\alpha_{j}-kn\right)\right)\\ -\eta_{i1}\left(\varepsilon \sigma +k\delta \sum_{j=1}^{n}\gamma_{j}-2\left(\delta^{2}+\sigma \right)\sum_{j=1}^{n}\eta_{j2}\right) \end{array}\right),$$

(19) $$\eta_{i2}=\frac{1}{2\sigma r}\left(k^{2}\gamma_{i}^{2}-\sigma \beta_{i}-4\eta_{i2}\left(k\delta \sum_{j=1}^{n}\gamma_{j}+\varepsilon \sigma +\left(\delta^{2}+\sigma \right)\left(\eta_{i2}-2\sum_{j=1}^{n}\eta_{j2}\right)\right)\right).$$

**Proof of Proposition** **1.** Substituting the optimal solutions from (12) and (13) into the HJB equation in (11) and simplifying yield

(20) $$\begin{array}{ll} rV_{i}^{NCA}\left(s\right) & =\frac{1}{2}\left({\left(k-\alpha_{i}\right)}^{2}-\beta_{i}s^{2}\right)+kF_{i}+\frac{k^{2}\gamma_{i}^{2}s^{2}}{2\sigma }-\frac{\sigma +\delta^{2}}{2\sigma }{\left(\frac{\partial V_{i}^{NCA}}{\partial s}\right)}^{2}\\ & +\frac{\partial V_{i}^{NCA}}{\partial s}\left(\sum_{j=1}^{n}\alpha_{j}+\sum_{j=1}^{n}\frac{\partial V_{j}^{NCA}}{\partial s}-\varepsilon s-kn-\frac{\delta }{\sigma }\left(ks\sum_{j=1}^{n}\gamma_{j}-\delta \sum_{j=1}^{n}\frac{\partial V_{j}^{NCA}}{\partial s}\right)\right) \end{array}$$

Differentiating (14) w.r.t. s, the following is obtained

(21) $$\frac{V_{i}^{NCA}\left(s\right)}{\partial s}=\eta_{i1}s+2\eta_{i2}s.$$

Substituting (14) and (21) into (20), then equating the coefficients of $\eta_{i0}+\eta_{i1}s+\eta_{i2}s^{2}$ in (20), the results in (15) and (16) are obtained. □

#### 4.1.2. Optimal Trajectory of the Pollutant Stock

The ordinary differential equation in (22) in the following is obtained by directly substituting (15) and (16) into (8)

(22) $${\dot{s}}^{NCA}(t)=C_{1}^{NCA}+\theta^{NCA}s^{NCA}(t),$$

where $C_{1}^{NCA}=\frac{\sigma +\delta^{2}}{\sigma }\sum_{j=1}^{n}\eta_{j1}+\sum_{j=1}^{n}\alpha_{j}-kn$ and $\theta^{NCA}=\frac{2\left(\sigma +\delta^{2}\right)}{\sigma }\sum_{j=1}^{n}\eta_{j2}-\frac{k\delta }{\sigma }\sum_{j=1}^{n}\gamma_{j}-\varepsilon$.

The optimal trajectory of the pollutant stock in (23) in the following is obtained by solving (22)

(23) $$s^{NCA}(t)=-\frac{C_{1}^{NCA}}{\theta^{NCA}}+C_{2}^{NCA}e^{t\theta^{NCA}},$$

where $C_{2}^{NCA}=s_{0}+\frac{C_{1}^{NCA}}{\theta^{NCA}}$, and $\theta^{NCA}<0$ ensures the stability of the steady state of the pollutant stock.

### 4.2. Symmetric Case (NCS)

#### 4.2.1. Solutions of the Model

**Remark** **1.** For the symmetric case, let $\alpha_{i}\;=\;\alpha$, $\beta_{i}\;=\;\beta$, $q_{i}\;=\;q$, $u_{i}\;=\;u$, $\gamma_{i}=\gamma$ and $F_{i}\;=\;F$ for i=1,2,⋯,n.

From (8) and (10), the HJB equation for any country can be written as

(24) $$rV^{NCS}=\underset{q,u}{\max}\left\{\pi +\frac{\partial V^{NCS}}{\partial s}\frac{ds(t)}{dt}\right\},$$

where $V^{NCS}=V^{NCS}\left(s\right)$ is the value function of the country.

The following optimal feedback production and abatement strategies of the country are obtained by taking the first-order conditions of the HJB equation in (24) w.r.t. q and u, respectively

(25) $$q^{NCS}=\frac{1}{\mu }\left(\alpha -k+n\frac{\partial V^{NCS}}{\partial s}\right),$$

(26) $$u^{NCS}=\frac{1}{\sigma }\left(k\gamma s-n\delta \frac{\partial V^{NCS}}{\partial s}\right).$$

**Proposition** **2.** In the symmetric non-cooperative differential game, the Nash equilibrium solutions of the value functions and instantaneous levels of production and abatement, represented by $V^{NCS}(s)$, $q^{NCS}(t)$ and $u^{NCS}(t)$, respectively, are

(27) $$V^{NCS}\left(s\right)=\eta_{0}+\eta_{1}s+\eta_{2}s^{2},$$

(28) $$q^{NCS}\;=\;\frac{1}{\mu }\left(\alpha -k+n\eta_{1}+2n\eta_{2}s\right),$$

(29) $$u^{NCS}\;=\;\frac{1}{\sigma }\left(k\gamma s-n\delta \left(\eta_{1}+2\eta_{2}s\right)\right),$$

where

(30) $$\eta_{0}=\frac{1}{r}\left(kF+\frac{1}{2}{\left(\alpha -k\right)}^{2}+n\eta_{1}\left(\alpha -k\right)+\frac{n^{2}\eta_{1}^{2}}{2\sigma }\left(\sigma +\delta^{2}\right)\right),$$

(31) $$\eta_{1}=\frac{1}{r}\left(2n\eta_{2}\left(\left(\alpha -k\right)+\frac{n\eta_{1}}{\sigma }\left(\sigma +\delta^{2}\right)\right)-\eta_{1}\left(\frac{nk\gamma \delta }{\sigma }+\varepsilon \right)\right),$$

(32) $$\eta_{2}=\frac{1}{2\sigma r}\left(k^{2}\gamma^{2}+4n^{2}\eta_{2}^{2}\left(\sigma +\delta^{2}\right)-4\eta_{2}\left(\varepsilon \sigma +nk\gamma \delta \right)-\beta \sigma \right).$$

**Proof of Proposition** **2.** Substituting the optimal solutions from (25) and (26) into the HJB equation in (24) and simplifying yield

(33) $$\begin{array}{ll} rV^{NCS}\left(s\right) & =kF+\frac{1}{2}\left({\left(\alpha -k\right)}^{2}-\beta s^{2}\right)+\frac{k^{2}\gamma^{2}s^{2}}{2\sigma }\\ & +\left(n\alpha -\varepsilon s-kn-\frac{nk\gamma \delta s}{\sigma }+\frac{n^{2}\left(\sigma +\delta^{2}\right)}{2\sigma }\frac{\partial V^{NCS}}{\partial s}\right)\frac{\partial V^{NCS}}{\partial s} \end{array}$$

Differentiating (27) w.r.t. s, the following is obtained

(34) $$\frac{\partial V^{NCS}}{\partial s}=\eta_{1}+2\eta_{2}s.$$

Substituting (27) and (34) into (33) and equating the coefficients of $\eta_{0}+\eta_{1}s+\eta_{2}s^{2}$ in (33), the results in (28) and (29) are obtained. □

#### 4.2.2. Optimal Trajectory of the Pollutant Stock

The ordinary differential equation in (35) in the following is obtained by substituting (28) and (29) into (8)

(35) $$\frac{\partial V^{NCS}}{\partial s}=\eta_{1}\;+\;2\eta_{2}s,$$

where $C_{1}^{NCS}=n\left(\alpha -k\right)+n^{2}\eta_{1}\left(1+\frac{\delta^{2}}{\sigma }\right)$ and $\theta^{NCS}=\frac{1}{\sigma }\left(2n^{2}\delta^{2}\eta_{2}-kn\gamma \delta \right)+2n^{2}\eta_{2}-\varepsilon$.

The optimal trajectory of the pollutant stock in (36) in the following can be obtained by solving (35)

(36) $$s^{NCS}(t)=\;-\frac{C_{1}^{NCS}}{\theta^{NCS}}+C_{2}^{NCS}e^{t\theta^{NCS}},$$

where $C_{2}^{NCS}\;=\;s_{0}+\frac{C_{1}^{NCS}}{\theta^{NCS}}$, and $\theta^{NCS}<0$ ensures the stability of the steady state of the pollutant stock.

## 5. Cooperative Differential Games

### 5.1. Asymmetric Case (CA)

#### 5.1.1. Solutions of the Model

The following HJB equation for country i is obtained from (8) and (10),

(37) $$rV^{CA}=\underset{q_{i},u_{i}}{\max}\left\{\sum_{i=1}^{n}\pi_{i}+\frac{\partial V^{CA}}{\partial s}\frac{ds(t)}{dt}\right\},$$

where $V^{CA}=V^{CA}\left(s\right)$ is the value function of country i.

The following optimal feedback production and abatement strategies for country i are obtained by taking the first-order conditions of the HJB equation in (37) w.r.t. $q_{i}$ and $u_{i}$, respectively

(38) $$q_{i}^{CA}=\frac{1}{\mu }\left(\alpha_{i}-k+\frac{\partial V^{CA}}{\partial s}\right),$$

(39) $$u_{i}^{CA}=\frac{1}{\sigma }\left(ks\gamma_{i}-\delta \frac{\partial V^{CA}}{\partial s}\right).$$

**Proposition** **3.** In the asymmetric cooperative differential game, the optimal solutions of the value functions and instantaneous levels of production and abatement, represented by $V^{CA}\left(s\right)$, $q_{i}^{CA}(t)$ and $u_{i}^{CA}(t)$, respectively, are

(40) $$V^{CA}\left(s\right)=\eta_{0}+\eta_{1}s+\eta_{2}s^{2},$$

(41) $$q_{i}^{CA}\;=\;\frac{1}{\mu }\left(\alpha_{i}-k+\eta_{1}+\eta_{2}s\right),$$

(42) $$u_{i}^{CA}\;=\;\frac{1}{\sigma }\left(ks\gamma_{i}-\delta \left(\eta_{1}+\eta_{2}s\right)\right),$$

where

(43) $$\eta_{0}=\frac{1}{r}\left(\begin{array}{l} k\sum_{j=1}^{n}F_{j}-\left(k-1\right)\sum_{j=1}^{n}\alpha_{j}+\frac{1}{2}\sum_{j=1}^{n}\alpha_{j}^{2}+kn\left(k-2\right)\\ -\left(1+\delta^{2}\right)\sum_{j=1}^{n}\eta_{j1}^{2}+\left(1+\frac{\delta^{2}}{\sigma }\right)\sum_{j=1}^{n}\eta_{j1} \end{array}\right),$$

(44) $$\eta_{1}=\frac{1}{r}\left(\begin{array}{l} \frac{2}{\sigma }\left(\sigma +\delta^{2}\right)\sum_{j=1}^{n}\eta_{j2}-2\left(1+\delta^{2}\right)\sum_{j=1}^{n}\eta_{j1}\eta_{j2}-\varepsilon \\ -\frac{k\delta }{\sigma }\left(\sum_{j=1}^{n}\gamma_{j}+\left(1-\sigma \right)\sum_{j=1}^{n}\gamma_{j}\eta_{j1}\right) \end{array}\right),$$

(45) $$\eta_{2}=\frac{1}{2\sigma r}\left(\begin{array}{l} 4k\delta \left(\sigma -1\right)\sum_{j=1}^{n}\gamma_{j}\eta_{j2}-k^{2}\left(\sigma -2\right)\sum_{j=1}^{n}\gamma_{j}^{2}\\ -\sigma \left(\sum_{j=1}^{n}\beta_{j}+4\left(1+\delta^{2}\right)\sum_{j=1}^{n}\eta_{j2}^{2}\right) \end{array}\right).$$

**Proof of Proposition** **3.** Substituting the optimal solutions in (41) and (42) into the HJB equation in (37) and simplifying yield

(46) $$\begin{array}{ll} rV^{CA}\left(s\right) & =\sum_{j=1}^{n}\alpha_{j}^{2}+k\left(\sum_{j=1}^{n}F_{j}-\sum_{j=1}^{n}\alpha_{j}+\frac{kn}{2}\right)-\frac{s^{2}}{2}\sum_{j=1}^{n}\beta_{j}-\frac{k^{2}s^{2}}{2\sigma }\left(\sigma -2\right)\sum_{j=1}^{n}\gamma_{j}^{2}\\ & +\left(\sum_{j=1}^{n}\alpha_{j}-kn-\varepsilon s+k\delta s\left(1-\frac{2}{\sigma }\right)\sum_{j=1}^{n}\gamma_{j}\;+\;\frac{n}{2}\left(1-\frac{\delta^{2}}{\sigma }\left(\sigma -2\right)\right)\frac{\partial V^{CA}}{\partial s}\right)\frac{\partial V^{CA}}{\partial s} \end{array}$$

Differentiating (40) w.r.t. s, the following is obtained

(47) $$\frac{\partial V^{CA}}{\partial s}\;=\;\eta_{1}\;+\;2\eta_{2}s.$$

Substituting (40) and (47) into (46) and equating the coefficients of $\eta_{0}+\eta_{1}s+\eta_{2}s^{2}$ in (46), the results in (41) and (42) are obtained. □

#### 5.1.2. Optimal Trajectory of the Pollutant Stock

The ordinary differential equation in (48) in the following is obtained by substituting (41) and (42) into (8)

(48) $${\dot{s}}^{CA}(t)=C_{1}^{CA}+\theta^{CA}s(t),$$

where $C_{1}^{CA}=\sum_{j=1}^{n}\alpha_{j}-kn+n\eta_{1}\left(1+\frac{\delta^{2}}{\sigma }\right)$ and $\theta^{CA}=2n\eta_{2}\left(1+\frac{\delta^{2}}{\sigma }\right)-\frac{k\delta }{\sigma }\sum_{j=1}^{n}\gamma_{j}-\varepsilon$.

The optimal trajectory of the pollutant stock in (49) in the following is obtained by solving (48)

(49) $$s^{CA}(t)=-\frac{C_{1}^{CA}}{\theta^{CA}}+C_{2}^{CA}e^{t\theta^{CA}},$$

where $C_{2}^{CA}=s_{0}+\frac{C_{1}^{CA}}{\theta^{CA}}$, and $\theta^{CA}<0$ ensures the stability of the steady state of the pollutant stock.

### 5.2. Symmetric Case (CS)

#### 5.2.1. Solutions of the Model

The HJB equation for the countries in (50) in the following is from (8) and (9) together with Remark 1

(50) $$rV^{CS}=\underset{q,u}{\max}\left\{n\pi +\frac{\partial V^{CS}}{\partial s}\frac{ds(t)}{dt}\right\},$$

where $V^{CS}=V^{CS}\left(s\right)$ is the value function of the countries.

The following optimal feedback strategies of production and abatement of the countries are obtained by taking the first-order conditions of the HJB equation in (50) w.r.t. q and u, respectively

(51) $$q^{CS}=\frac{1}{\mu }\left(\alpha -k+\frac{\partial V^{CS}}{\partial s}\right),$$

(52) $$u^{CS}=\frac{1}{\sigma }\left(k\gamma s-\delta \frac{\partial V^{CS}}{\partial s}\right).$$

**Proposition** **4.** In the symmetric cooperative differential game, the optimal solutions of the value functions and instantaneous levels of production and abatement, represented by $V^{CS}(s)$, $q^{CS}(t)$ and $u^{CS}(t)$, respectively, are

(53) $$V^{CS}\left(s\right)=\eta_{0}+\eta_{1}s+\eta_{2}s^{2},$$

(54) $$q^{CS}=\frac{1}{\mu }\left(\alpha -k+\eta_{0}+\eta_{1}s+\eta_{2}s^{2}\right),$$

(55) $$u^{CS}\;=\;\frac{1}{\sigma }\left(k\gamma s-\delta \left(\eta_{0}\;+\;\eta_{1}s+\;\eta_{2}s^{2}\right)\right),$$

where

(56) $$\eta_{0}=\frac{n}{r}\left(kF+\frac{1}{2}{\left(k-\alpha \right)}^{2}-\eta_{1}\left(k-\alpha \right)+\frac{\eta_{1}^{2}}{2}\left(1+\frac{\delta^{2}}{\sigma }\right)\right),$$

(57) $$\eta_{1}=\frac{1}{\sigma r}\left(2n\eta_{2}\left(\eta_{1}\left(\sigma +\delta^{2}\right)-\sigma \left(k-\alpha \right)\right)-\eta_{1}\left(kn\gamma \delta +\varepsilon \sigma \right)\right),$$

(58) $$\eta_{2}=\frac{n}{2\sigma r}\left(k^{2}\gamma^{2}-\beta \sigma \right)-\frac{2\eta_{2}}{\sigma r}\left(kn\gamma \delta +\varepsilon \sigma +n\eta_{2}\left(\sigma +\delta^{2}\right)\right).$$

**Proof of Proposition** **4.** Substituting the optimal solutions of (51) and (52) into the HJB equation in (50) and simplifying yield

(59) $$\begin{array}{ll} rV^{CS}\left(s\right) & =\frac{1}{2\sigma }nk^{2}\gamma^{2}s^{2}+\frac{n}{2}\left(2kF+{\left(k-\alpha \right)}^{2}-s^{2}\beta \right)\\ & +\left(n\alpha -\varepsilon s-\frac{kn}{\sigma }\left(\gamma \delta s+\sigma \right)+\frac{n}{2\sigma }\left(\sigma +\delta^{2}\right)\frac{\partial V^{CS}}{\partial s}\right)\frac{\partial V^{CS}}{\partial s} \end{array}$$

Differentiating (53) w.r.t. s, the following is obtained

(60) $$\frac{\partial V^{CS}}{\partial s}=\eta_{1}+2\eta_{2}s.$$

Substituting (53) and (60) into (59) and equating the coefficients of $\eta_{0}+\eta_{1}s+\eta_{2}s^{2}$ in (59), the results in (54) and (55) are obtained. □

#### 5.2.2. Optimal Trajectory of the Pollutant Stock

The ordinary differential equation in (61) in the following is obtained by substituting (54) and (55) into (8)

(61) $${\dot{s}}^{CS}(t)=C_{1}^{CS}+\theta^{CS}s^{CS}(t),$$

where $C_{1}^{CS}=2\mu \left(\alpha -k+n\eta_{1}\left(1+\frac{\delta^{2}}{\sigma }\right)\right)$ and $\theta^{CS}=\frac{1}{\sigma }kn\gamma \delta +\varepsilon -2n^{2}\eta_{2}\left(1+\frac{\delta^{2}}{\sigma }\right)$.

The optimal trajectory of the pollutant stock in (62) in the following is obtained by solving (61)

(62) $$s^{CS}(t)=-\frac{C_{1}^{CS}}{\theta^{CS}}+C_{2}^{CS}e^{t\theta^{CS}},$$

where $C_{2}^{CS}=s_{0}+\frac{C_{1}^{CS}}{\theta^{CS}}$, and $\theta^{CS}<0$ ensures the stability of the steady state of the pollutant stock.

## 6. Numerical Simulation

In the following, numerical results of the case of a duopoly game, i.e., n= 2, are demonstrated. The effects of the initial allocations of pollution emission permits $F_{1}$ and $F_{2}$ (F) and the pollution emission coefficient μ on the value functions $V_{1}$ and $V_{2}$ (V), on the production levels $q_{1}^{*}$ and $q_{2}^{*}$, on the abatement efforts $u_{1}^{*}$ and $u_{2}^{*}$ ($u^{*}$), and on the pollutant stock levels s(t) are studied through numerical simulation.

The selection of important parameter values in the models is similar to that in List and Mason [21]. The following example comes from global warming and U.S. carbon emissions (List and Mason, 2001). Nordhaus [33] derived that the carbon decay rate in the atmosphere per year is about 1%. Therefore, ε=0.01 is used in the numerical study. He also estimated that the discount rate ranges from 1% to 7%. Hence, r=0.02 is used in the numerical study. According to the ICE End of Day Reports (https://www.theice.com/market-data/end-of-day-reports), k=21 dollars per ton is used. Based on empirical evidences [21,34,35,36,37], $\beta_{1}=0.0051$, $\beta_{2}=0.005$, $\alpha_{1}=27$, $\alpha_{2}=28$, $\gamma_{1}=0.000001$, $\gamma_{2}=0.0000011$, δ=2, σ=0.1, μ=0.01, s(0)=167 (billion tons), $F_{1}=56$ (billion tons) and $F_{2}=51$ (billion tons) are used. In the *NCS* and *CS* scenarios, β=0.0051, α=27, γ=0.000001, and F=56 are used. In all the plots in the following, t varies from 0 to 200 with an increment of 1.

The optimal solutions of the value functions, instantaneous levels of production and abatement, and the optimal trajectories of the pollutant stock levels are obtained by substituting the above parameter values into (14)–(16) and (23) for NCA, (27)–(29) and (36) for NCS, (40)–(42) and (49) for CA and (53)–(55) and (62) for CS, respectively. After simplification, these optimal solutions are presented in the following.

**Case** *NCA*

$s^{NCA}(t)=8.932+158.068e^{-0.522583t}$, $q_{1}^{NCA}(t)=589.56-0.633s(t)$,
$q_{2}^{NCA}(t)=690.117-0.615s(t)$, $u_{1}^{NCA}(t)=2.088+0.127s(t)$,
$u_{2}^{NCA}(t)=1.977+0.123s(t)$, $V_{1}^{NCA}\left(s\right)=1193.29-0.104s(t)-0.00317s^{2}(t)$,
$V_{2}^{NCA}\left(s\right)=1094.84-0.099s(t)-0.00307s^{2}(t)$.

**Case** *NCS*

$s^{NCS}(t)=8.29039+158.71e^{-0.519944t}$, $q^{\mathrm{NCS}}(t)=589.56-0.633s(t)$,
$u^{\mathrm{NCS}}(t)=1.876+0.124s(t)$, $V^{\mathrm{NCS}}\left(s\right)=1193.42-0.094s(t)-0.0031s^{2}(t)$.

**Case** *CA*

$s^{CA}(t)=0.485+166.515e^{-0.900294t}$, $q_{1}^{CA}(t)=584.678-1.08465s(t)$,
$q_{2}^{CA}(t)=684.678-1.085s(t)$, $u_{1}^{CA}(t)=2.088+0.127s(t)$,
$u_{2}^{CA}(t)=3.064+0.217s(t)$, $V^{CA}\left(s\right)=2288.47-0.153s(t)-0.005423s^{2}(t)$.

**Case** *CS*

$s^{CS}(t)=0.451+166.549e^{-0.895778t}$, $q^{CS}(t)=585.859-1.079s(t)$,
$u^{CS}(t)=2.828+0.216s(t)$, $V^{CS}\left(s\right)=2387.12-0.141s(t)-0.005396s^{2}(t)$.

### 6.1. The Optimal Pollutant Stock Levels

**Figure 1:**
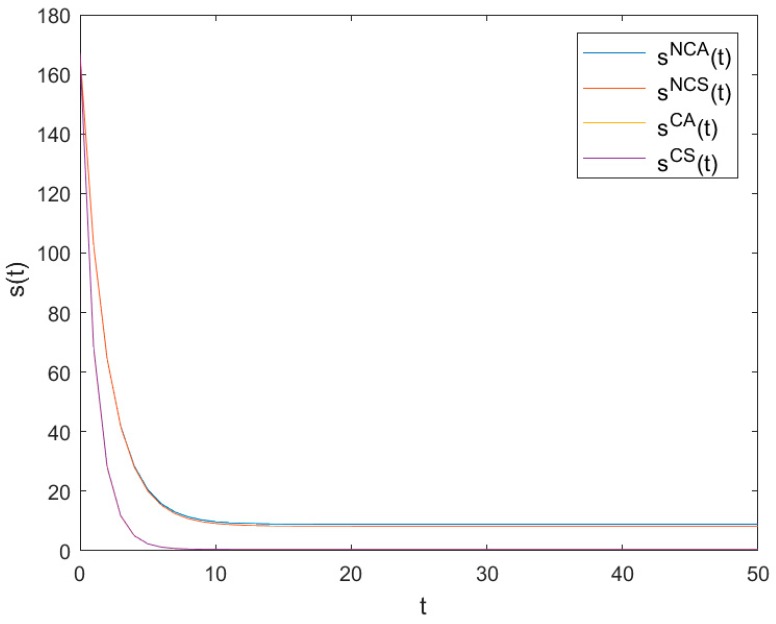
Evolutions of optimal trajectories of the pollutant stock levels.

Figure 1 shows the optimal evolution trajectories of pollutant stock levels of *NCA*, *NCS*, *CA* and *CS* as time t changes. It can be seen from Figure 1 that all pollutant stock levels decrease quickly at the beginning and finally reach low steady levels $s^{\mathrm{NCA}}$, $s^{\mathrm{NCS}}$, $s^{\mathrm{CA}}$ and $s^{\mathrm{CS}}$, respectively, with $s^{\mathrm{NCA}}=8.932$, $s^{\mathrm{NCS}}=8.29039$, $s^{\mathrm{CA}}=0.485$ and $s^{\mathrm{CS}}=0.451$.

Obviously, $s^{\mathrm{NCA}}>s^{\mathrm{NCS}}>s^{\mathrm{CA}}>s^{\mathrm{CS}}$ holds, which means that the pollutant stock levels in symmetric cases are better than those in asymmetric cases, and those in cooperative cases are better than those in non-cooperative cases. It further shows that both symmetry and cooperation are helpful in reducing pollutant stock levels.

### 6.2. The Optimal Production Levels

**Figure 2:**
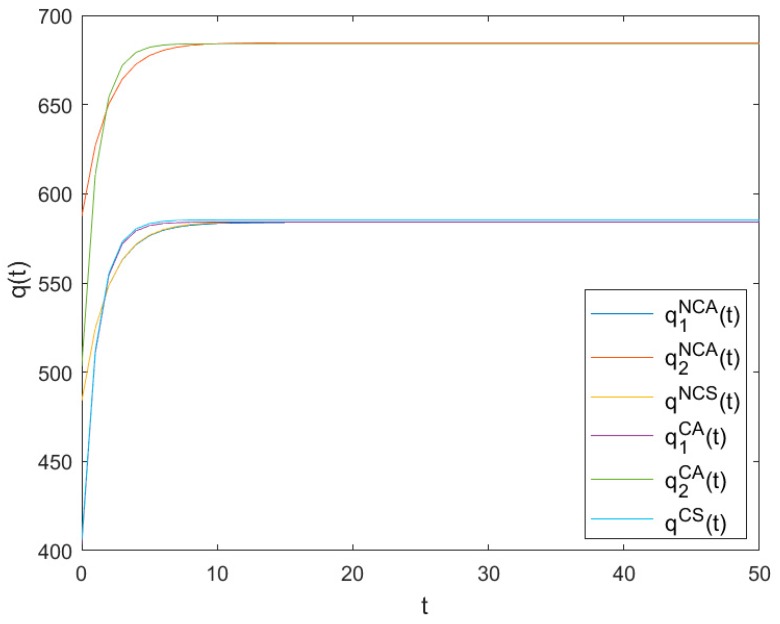
Evolutions of the optimal production levels.

Figure 2 shows the evolutionary trajectories of the optimal production levels of *NCA*, *NCS*, *CA* and *CS* as time t changes. It can be seen from Figure 2 that all optimal production levels increase quickly in the beginning and finally reach high steady levels $q_{1}^{\mathrm{NCA}}$, $q_{2}^{\mathrm{NCA}}$, $q^{\mathrm{NCS}}$ $q_{1}^{\mathrm{CA}}$, $q_{2}^{\mathrm{CA}}$ and $q^{\mathrm{CS}}$, respectively, with $q_{1}^{\mathrm{NCA}}=583.906$, $q_{2}^{\mathrm{NCA}}=684.624$, $q^{\mathrm{NCS}}=585.475$, $q_{1}^{\mathrm{CA}}=584.153$, $q_{2}^{\mathrm{CA}}=684.153$ and $q^{\mathrm{CS}}=585.372$.

Apparently, $q_{1}^{\mathrm{NCA}}+q_{2}^{\mathrm{NCA}}>2q^{\mathrm{NCS}}$ and $q_{1}^{\mathrm{CA}}+q_{2}^{\mathrm{CA}}>2q^{\mathrm{CS}}$ mean that both the optimal production levels and the pollution emission levels in asymmetric games are more significant than those in symmetric games. Furthermore, $q_{1}^{\mathrm{NCA}}+q_{2}^{\mathrm{NCA}}>q_{1}^{\mathrm{CA}}+q_{2}^{\mathrm{CA}}$ and $q^{\mathrm{NCS}}>q^{\mathrm{CS}}$ show that both the optimal production levels and the pollution emission levels in non-cooperative games are higher than those in cooperative games. It further shows that both symmetry and cooperation are helpful in reducing pollution emission levels.

### 6.3. The Optimal Pollution Abatement Levels

**Figure 3:**
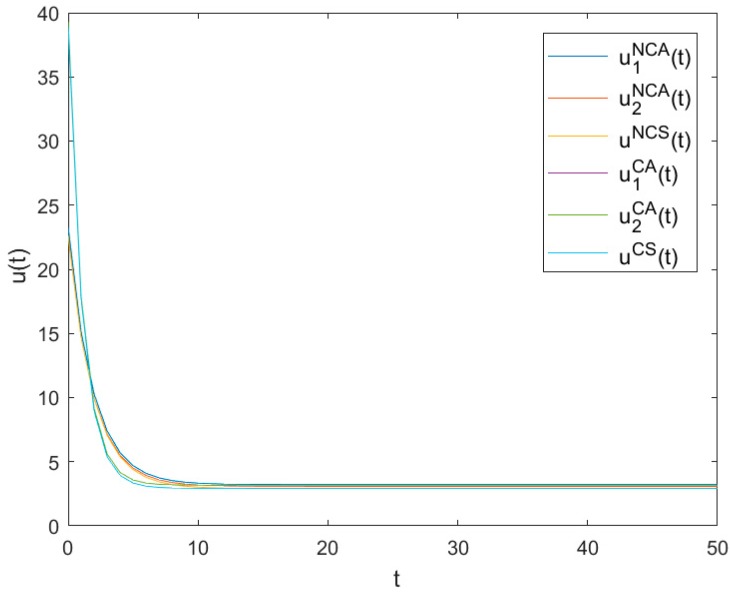
Evolutions of the optimal pollution abatement levels.

Figure 3 shows the evolutionary trajectories of the optimal pollution abatement levels of *NCA*, *NCS*, *CA* and *CS* as time t changes. It can be seen from Figure 3 that all pollution abatement levels decrease quickly at the beginning and finally reach low steady levels $u_{1}^{\mathrm{NCA}}$, $u_{2}^{\mathrm{NCA}}$, $u^{\mathrm{NCS}}$, $u_{1}^{\mathrm{CA}}$, $u_{2}^{\mathrm{CA}}$ and $u^{\mathrm{CS}}$, respectively, with $u_{1}^{\mathrm{NCA}}=3.2207$, $u_{2}^{\mathrm{NCA}}=3.07729$, $u^{\mathrm{NCS}}=2.90665$, $u_{1}^{\mathrm{CA}}=3.16955$, $u_{2}^{\mathrm{CA}}=3.16956$ and $u^{\mathrm{CS}}=2.92573$.

Apparently, $u_{1}^{\mathrm{NCA}}+u_{2}^{\mathrm{NCA}}>2u^{\mathrm{NCS}}$ and $u_{1}^{\mathrm{CA}}+u_{2}^{\mathrm{CA}}>2u^{\mathrm{CS}}$ mean that both the optimal pollution abatement levels in asymmetric games are more significant than those in symmetric games. $u^{\mathrm{NCS}}<u^{\mathrm{CS}}$ and $u_{1}^{\mathrm{NCA}}+u_{2}^{\mathrm{NCA}}<u_{1}^{\mathrm{CA}}+u_{2}^{\mathrm{CA}}$ show that both the optimal pollution abatement levels in non-cooperative games are smaller than those in cooperative games. It further shows that symmetry goes against pollution abatement effort levels and cooperation is helpful to pollution abatement effort levels.

### 6.4. The Optimal Value Functions

**Figure 4:**
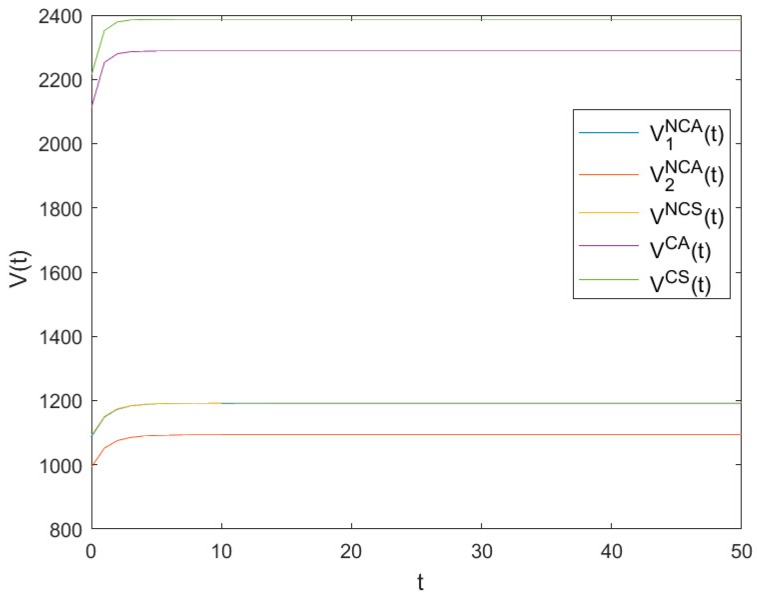
Evolutions of the optimal value functions.

Figure 4 shows the evolutionary trajectories of the optimal value functions of *NCA*, *NCS*, *CA* and *CS* as time t changes. It can be seen from Figure 4 that all value functions increase quickly in the beginning and finally reach high steady levels $V_{1}^{\mathrm{NCA}}$, $V_{2}^{\mathrm{NCA}}$, $V^{\mathrm{NCS}}$ $V_{1}^{\mathrm{CA}}$, $V_{2}^{\mathrm{CA}}$, and $V^{\mathrm{CS}}$, respectively, with $V_{1}^{\mathrm{NCA}}=1193.29$, $V_{2}^{\mathrm{NCA}}=1094.84$, $V^{\mathrm{NCS}}=1193.42$, $V^{\mathrm{CA}}=2288.47$ and $V^{\mathrm{CS}}=2387.12$.

Apparently, $V_{1}^{\mathrm{NCA}}+V_{2}^{\mathrm{NCA}}<2V^{\mathrm{NCS}}$ and $V^{\mathrm{CA}}<V^{\mathrm{CS}}$ mean that both the optimal value functions in asymmetric games are smaller than those in symmetric games. Furthermore, $V_{1}^{\mathrm{NCA}}+V_{2}^{\mathrm{NCA}}<V^{\mathrm{CA}}$ and $V^{\mathrm{NCS}}<V^{\mathrm{CS}}$ show that both the optimal value functions in non-cooperative games are smaller than those in cooperative games. It further shows that both symmetry and cooperation are helpful to the value functions. 

## 7. Dividends Analysis

### 7.1. Cooperative Dividends

**Definition** **1.** The cooperative dividend is the difference between optimal payoffs of the cooperative game and the non-cooperative game.

**Definition** **2.** A cooperative agreement is feasible if and only if the total optimal cooperative payoffs are greater than the sum of the individual non-cooperative payoffs.

The so-called agreement feasibility refers to the collective rational choice of many parties. Definition 2 shows that a cooperative agreement is feasible if sum of individual payoffs of the cooperation strategy is more than that of the current non-cooperative strategy.

**Figure 5:**
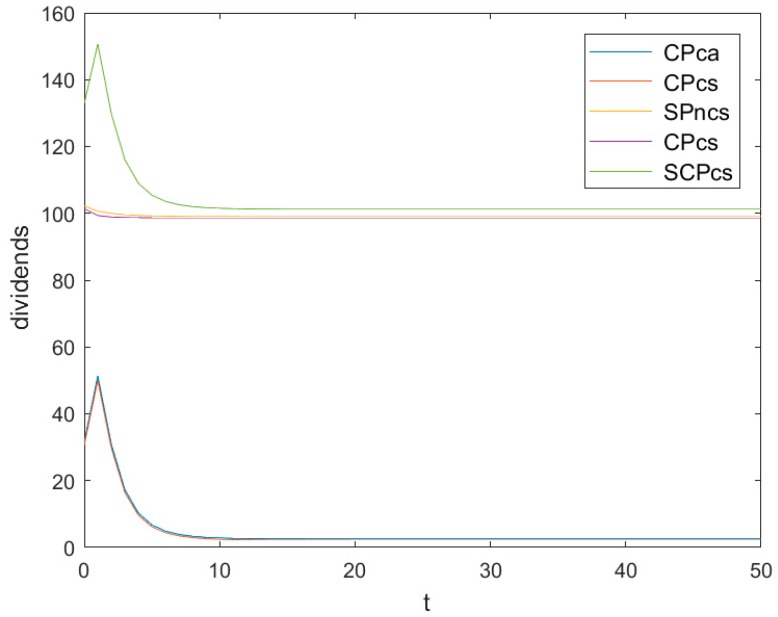
Evolutions of different optimal dividends.

Figure 5 shows the evolutionary trajectories of the different optimal dividend levels as time t changes, in which *CPsc* denotes the cooperative dividend of a symmetric game, *CPca* denotes the cooperative dividend of an asymmetric game, *CPcs* denotes the symmetric dividend of a cooperative game, *CPncs* denotes the symmetric dividend of a non-cooperative game, and *SCPcs* denotes the cooperative and symmetric dividend of a game.

#### 7.1.1. The Cooperative Dividend of an Asymmetric Game

**Definition** **3.** The cooperative dividend of an asymmetric game is the difference between optimal payoffs of its asymmetric cooperative scenario and its asymmetric non-cooperative scenario.

According to Definition 3, the cooperative dividend of an asymmetric game can be written as

$$\begin{array}{ll} CPca= & V^{CA}-(V_{1}^{NCA}+V_{2}^{NCA})\\ & =2.5804-150.372e^{-1.80059t}+155.917e^{-1.04517t}-26.3881e^{-0.9200294t}+49.7445e^{-0.522583t} \end{array}.$$

Figure 5 also shows that the cooperative dividend of an asymmetric game decreases quickly at the beginning and finally reaches a low steady level $CPca^{\infty }=\underset{t\to \infty }{\lim}CPca=2.5804$.

#### 7.1.2. The Cooperative Dividend of a Symmetric Game

**Definition** **4.** The cooperative dividend of a symmetric game is the difference between optimal payoffs of its symmetric cooperative scenario and its symmetric non-cooperative scenario.

According to Definition 4, the cooperative dividend of a symmetric game can be written as

$$\begin{array}{ll} CPsc & =V^{CS}-2V^{NCS}\\ & =2.20858-149.676e^{-1.79156t}+156.386e^{-1.03989t}-24.3631e^{-0.895778t}+46.1037e^{-0.519944t} \end{array}.$$

Figure 5 also shows that the cooperative dividend of a symmetric game decreases quickly at the beginning and finally reaches a low steady level $CPsc^{\infty }=\underset{t\to \infty }{\lim}CPca=2.20858$.

### 7.2. Symmetric Dividends

**Definition** **5.** The symmetric dividend is the difference between optimal payoffs of the symmetric game and the asymmetric game.

**Definition** **6.** A symmetric agreement is stable if and only if the total optimal symmetric payoffs are greater than the total optimal asymmetric payoffs.

#### 7.2.1. The Symmetric Dividend of a Non-Cooperative Game

**Definition** **7.** The symmetric dividend of a non-cooperative game is the difference between optimal payoffs of its symmetric non-cooperative scenario and its asymmetric non-cooperative scenario.

According to Definition 7, the symmetric dividend of a non-cooperative game can be written as

$$\begin{array}{ll} CPnsc & =2V^{NCS}-(V_{1}^{NCA}+V_{2}^{NCA})\\ & =99.0347+155.917e^{-1.04517t}-156.386e^{-1.03989t}+49.7445e^{-0.522583t}-46.1037e^{-0.519944t} \end{array}.$$

Figure 5 also shows that the symmetric dividend of a non-cooperative game decreases quickly at the beginning and finally reaches a low steady level $CPnsc^{\infty }=\underset{t\to \infty }{\lim}CPnsc=99.0347$.

#### 7.2.2. The Symmetric Dividend of a Cooperative Game

**Definition** **8.** The symmetric dividend of a cooperative game is the difference between optimal payoffs of its symmetric cooperative scenario and its asymmetric cooperative scenario.

According to Definition 8, the symmetric dividend of a cooperative game can be written as

$$\begin{array}{ll} CPcs & =V^{CS}-V^{CA}\\ & =98.6629+150.372e^{-1.80059t}-149.676e^{-1.79156t}+26.3881e^{-0.900294t}-24.3631e^{-0.895778t} \end{array}.$$

Figure 5 also shows that the symmetric dividend of a cooperative game decreases quickly at the beginning and finally reaches a low steady level $CPcs^{\infty }$ $=\underset{t\to \infty }{\lim}CPcs=98.6629$.

### 7.3. The Cooperative Symmetric Dividend of a Game

**Definition** **9.** The cooperative symmetric dividend of a game is the difference between optimal payoffs of its symmetric non-cooperative scenario and its asymmetric cooperative scenario.

**Definition** **10.** A cooperative symmetric agreement is stable if and only if the sum of the optimal cooperative symmetric payoffs is greater than the sum of the optimal asymmetric non-cooperative payoffs.

According to Definition 9, the cooperative symmetric dividend of a game can be written as

$$\begin{array}{ll} SCPcs & =V^{CS}-(V_{1}^{NCA}+V_{2}^{NCA})\\ & =101.243-149.676e^{-1.79156t}+155.917e^{-1.04517t}-24.3631e^{-0.895778t}+49.7445e^{-0.522583t} \end{array}.$$

Figure 5 also shows that the cooperative symmetric dividend of a game decreases quickly at the beginning and finally reaches a low steady level $SCPcs^{\infty }=\underset{t\to \infty }{\lim}SCPcs=101.243$.

Obviously, $SCPcs^{\infty }>CPnsc^{\infty }>CPcs^{\infty }>CPca^{\infty }>CPsc^{\infty }$ holds, which means that the cooperative symmetric dividend of a game is the largest and the cooperative dividend of a symmetric game is the smallest.

## 8. Policies and Managerial Implications

Results mentioned above show that symmetry and cooperation are not only helpful in reducing both pollutant stock levels and pollutant emission levels but also helpful to payoffs of all countries. These results also prove the Paris agreement’s idea which is that as each country implements its pledge, others can learn what is feasible, and that collaborative global climate protection will emerge. So, we can preliminarily reach the following five necessary judgments.

First, the competition between countries is integral and an essential strategic factor. A country should consciously put competition factors into consideration in its global strategy.

Second, in global competition, a country needs to build its comparative advantage, which is the basis of cooperation. Only by taking the initiative in the competition can it better carry out international cooperation.

Third, from the perspective of global governance, the compromise between countries in international competition does not necessarily mean clandestine collusion against the value of other countries. On the contrary, it is likely to lead to a more effective allocation of global resources.

Fourth, the formation of regional alliances similar to the European Union will help in improving the symmetry of participants, and also be more conducive to production efficiency and pollution reduction.

At last, the future global climate agreements following the Kyoto Protocol and the Paris Climate Agreement should strive to enhance cooperation, symmetry, and transparency in international production and pollution reduction. 

## 9. Conclusions

In this study, infinite-horizon cooperative and non-cooperative differential games are proposed to describe strategies of production planning and pollution abating of n countries under cap-and-trade regulations. Their asymmetric and symmetric feedback solutions are obtained using HJB equations.

These countries in the game simultaneously make two decisions—how much product to produce and how much pollution to abate under cap-and-trade regulations. In other words, each country makes decisions about how much pollution to abate or how much pollution emission right to buy or sell when deciding how much product to produce. Furthermore, the equilibrium solutions of the value functions, instantaneous levels of production and abatement, and the optimal trajectories of the pollutant stock levels vary at the beginning and finally reach their steady levels.

Both symmetry and cooperation are shown to have significant impacts on the production planning and pollution abating decisions. These impacts are:

1. Both symmetry and cooperation are helpful in reducing pollutant stock levels, abating pollution emission levels, and improving value functions.
2. Dividends increase in the following order: The cooperative dividend of a symmetric game, the cooperative dividend of an asymmetric game, the symmetric dividend of a cooperative game, the symmetric dividend of a non-cooperative game, and the cooperative and symmetric dividend of a game.

As concluded above, the cooperation of production plan and pollution emission significantly affect the decisions of the countries. However, how to share their information and how to improve their production cooperation among the n countries are interesting research topics. Moreover, equilibria mentioned above may vibrate under uncertain circumstances.

The following extensions are of interest for future research:

1. The deterministic differential games are based on assumptions of specific environmental damages which make the problem tractable. Further development is to consider uncertain environmental damages for which a stochastic differential game will be appropriate.
2. The ordinary differential games are based on the assumption that the environmental damage cost depends on the total pollutant stock level without considering its spatial diffusion. Partial differential games may be employed to consider the spatial diffusion.
3. The differential games are only constrained by the dynamical pollutant stock levels. If the pollution emission permits can be traded with dynamical prices, two or more dynamical constraints may be considered as shown in Xin and Sun [38].

## Figures and Tables

**Table 1:** Some game models of transboundary emissions of CO_2_/pollution.

Study	Objective Function	Stock Dynamics	Game Type
Long (1992)	$\int_{0}^{\infty }e^{-rt}\left(R_{i}(e_{i}(t))-D_{i}(s(t))\right)dt$	$\frac{ds(t)}{dt}=\sum_{i=1}^{2}e_{i}-\varepsilon s(t)$	Noncooperative
List and Mason (2001)	$\int_{0}^{\infty }e^{-rt}\left(R_{i}(e_{i}(t))-D_{i}(s(t))\right)dt$	$\frac{ds(t)}{dt}=\sum_{i=1}^{2}e_{i}-\varepsilon s(t)$	Cooperative, Noncooperative
Yeung (2007)	$\int_{0}^{T}e^{-rt}\left(R_{i}(q_{i}(t))-G_{i}(u_{i}(t))-D_{i}(q_{i}(t),s(t))\right)dt$	$\frac{ds(t)}{dt}=\sum_{i=1}^{2}\mu_{i}q_{i}\left(t\right)-\sum_{j=1}^{2}\gamma_{j}u_{j}\left(t\right)\sqrt{s\left(t\right)}-\varepsilon s\left(t\right)$	Cooperative
Masoudi and Zaccour (2013)	$\int_{0}^{\infty }e^{-rt}\left(R_{i}(e_{i}(t))-D_{i}(s(t))\right)dt$	$\frac{ds(t)}{dt}=\mu \sum_{i=1}^{2}e_{i}-\varepsilon s(t)$	Cooperative, Noncooperative
Bertinelli, Camacho and Zou (2014)	$\int_{0}^{T}e^{-rt}\left(-G_{i}(s_{i}(t),u_{i}(t))-D_{i}(s(t))\right)dt+e^{-rT}S\left(s(T)\right)$	$\frac{ds(t)}{dt}=\sum_{i=1}^{2}e_{i}+\mu \sum_{i=1}^{2}u_{i}-\varepsilon s(t)$	Non-cooperative
Li (2014)	$\int_{0}^{\infty }e^{-rt}\left(R_{i}(e_{i}(t))+M_{i}(e_{i}(t))-D_{i}(s(t))\right)dt$	$\frac{ds(t)}{dt}=\sum_{i=1}^{2}e_{i}-\varepsilon s(t)$	Cooperative, Noncooperative
Gromova and Plekhanova (2015)	$\int_{0}^{\infty }e^{-rt}\left(R_{i}(e_{i}(t))-D_{i}(s(t))\right)dt$	$\frac{ds(t)}{dt}=\mu \sum_{i=1}^{2}e_{i}-\varepsilon s(t)$	Cooperative, Noncooperative
Benchekroun and Martín-Herrán (2015)	$\int_{0}^{\infty }e^{-rt}\left(R_{i}(e_{i}(t))-D_{i}(s(t))\right)dt$	$\frac{ds(t)}{dt}=\sum_{i=1}^{2}e_{i}-\varepsilon s(t)$	Non-cooperative
Huang, He and Hua (2015)	$\int_{0}^{T}e^{-rt}\left(R_{i}(q_{i}(t))-G_{i}(u_{i}(t))-D_{i}(s(t))\right)dt-e^{-rT}S\left(s(T)\right)$	$\frac{ds(t)}{dt}=\sum_{i=1}^{2}q_{i}\left(t\right)-\sum_{j=1}^{2}\gamma_{j}u_{j}\left(t\right)\sqrt{s\left(t\right)}-\varepsilon s\left(t\right)$	Cooperative, Noncooperative
This study	$\int_{0}^{\infty }e^{-rt}\left(R_{i}(q_{i}(t),s(t),u_{i}(t))-G_{i}(u_{i}(t))-D_{i}(s(t))\right)dt$	$\frac{ds(t)}{dt}=\mu \sum_{i=1}^{n}q_{i}\left(t\right)-\delta \sum_{i=1}^{n}u_{i}\left(t\right)-\varepsilon s\left(t\right)$	Cooperative, Noncooperative

Note that notations in Table 1 are defined in Section 3.

**Table 2:** Notations.

Symbols	Descriptions
$q_{i}(t)$	the quantity of goods produced by country i in time t
$e_{i}(t)$	the amount of pollutant emitted by country i in time t
μ	a pollution emission coefficient which represents the current technology level of clean production, μ>0
$R_{i}^{\prime }(t)$	the net revenue function of country i in time t, which is strictly concave and quadratic, and depends on its pollution emission amount (List and Mason, 2001; Breton et al., 2005; Yeung, 2007; Li, 2014)
$\alpha_{i}$	a positive constant for country i
$D_{i}(s(t))$	the environmental damage cost to country i in time t
$\beta_{i}$	a positive scaling parameter for country i
s(t)	a pollutant stock level in time t contributed by the emissions of the n countries
$u_{i}(t)$	the abatement effort of country i in time t
$G_{i}(u_{i}(t))$	the pollution abatement cost function of country i in time t
σ	a positive scaling parameter
δ	a positive scaling parameter, δ>0
$B_{i}\left(u_{i}(t),s(t)\right)$	the egoistic part of pollution abatement amount of country i in time t
$\gamma_{i}$	a positive constant.
$F_{i}$	the initial allocation of pollution emission permit of country i
k(t)	the emission permit trading price in time t
$R_{i}^{\prime \prime }\left(\cdot \right)$	the trading revenue of country i
ε	a natural absorption rate of pollutants, ε>0
